# Comparison of Radiofrequency Ablation and Microwave Ablation for the Management of Hepatocellular Carcinoma: A Systematic Review and Meta-Analysis of Randomized Controlled Trials

**DOI:** 10.7759/cureus.67938

**Published:** 2024-08-27

**Authors:** Tariq M Shaqran, Jaber Alharbi, Shahad K Al-Hunbusi, Rayan A Alharbi, Mayadah Alawaji, Aman M Diqarshawi, Rakan J Almokhlef, Alanoud A Alfaqih, Ruyuf A Alhumaidi, Hussam A Alzahrani, Ibtesam M Alzyad, Zainab S Alwusaybie, Nashmi M Alotaibi, Nawaf Jamaan Alzahrani

**Affiliations:** 1 Family Medicine, King Salman Armed Forces Hospital, Tabuk, SAU; 2 College of Medicine, Qassim University, Qassim, SAU; 3 College of Medicine, Taif University, Taif, SAU; 4 Family Medicine, Qassim University, Qassim, SAU; 5 Faculty of Medicine, Qassim University, Qassim, SAU; 6 Radiology, East Jeddah General Hospital, Jeddah, SAU; 7 Medicine, King Saud Bin Abdulaziz University for Health Sciences, Riyadh, SAU; 8 College of Medicine, Imam Mohammad Ibn Saud Islamic University, Riyadh, SAU; 9 College of Medicine, King Khalid University, Abha, SAU; 10 College of Medicine, King Faisal University, Al Ahsa, SAU; 11 Medicine and Surgery, Al Baha University, Al Baha, SAU

**Keywords:** carcinoma, microwave ablation, ablative therapies, radiofrequency ablation, hepatocellular carcinoma

## Abstract

Hepatocellular carcinoma (HCC) is a common critical type of hepatic cancer worldwide. Recent guidelines have considered ablative therapeutic approaches as the primary option for managing early-stage surgically untreatable HCC. Among these therapies, radiofrequency ablation (RFA) and microwave ablation (MWA) have attained a significant role due to their efficacy and theoretical advantages. This review aims to compare and analyze the efficacy and safety of two common modalities, i.e., MWA and RFA, in the management of HCC. The literature search included PubMed, Cochrane Central Register of Controlled Trials, Medline, and Ovid for articles published until 2024. The outcomes included the local tumor progression (LTP), complete ablation (CA), the overall survival (OS) rate, or major complications. A meta-analysis was performed using Review Manager 5.3. The systematic review included six randomized controlled trials, including 826 patients. The findings revealed that MWA resulted in lower LTP and higher CA rates compared to RFA. However, the effect of complications was higher in the MWA therapy group. Despite that, the differences between all parameters were not significant. Statistical significance was not evident in the OS rates between the two modalities. Three studies found comparable survival rates between the two modalities, while one study reported similar local tumor recurrence-free survival rates between the two approaches. Both techniques appear to be effective and safe for the management of liver tumors, providing clinicians with valuable options for personalized patient care. Further high-quality research is needed to confirm these findings and guide clinical decision-making.

## Introduction and background

Hepatocellular carcinoma (HCC) is a common critical type of hepatic carcinoma worldwide. Globally, it constitutes around 85%-90% of all primary liver malignancies [1]. The prevalence of HCC is a global burden. The mortality rate to incidence ratio is 0.95 for HCC, with only a 6.9% five-year survival rate. There are large differences in the geographical incidences and mortalities [2]. Worldwide, about 782,000 malignancies are predicted to occur globally, resulting in about 600,000 deaths annually. It is expected to be the fifth most prevalent malignant tumor among males and seventh among females [3]. In Saudi Arabia, most patients with HCC receive diagnosis at mid or late stages, with poor prognosis and limited treatment options available [4].

Undoubtedly, one of the major steps for preventing HCC and improving its prognosis is to ensure early diagnosis. Early diagnosis and staging accuracy are particularly important in the efficient management of HCC. Recent guidelines have emphasized the importance of imaging techniques in diagnosing and staging the disease to ensure appropriate treatment planning and management [5]. However, many cases of HCC are detected at later stages where curative surgeries cannot be performed which results in alternative treatments being used [6].

Recently, ablative therapeutic approaches have been considered the primary option for managing early-stage surgically untreatable HCC. Among these therapies, radiofrequency ablation (RFA) has attained a significant role due to its efficacy, with a five-year survival rate of 40%-70% with improved safety and low recurrence rates. In addition, thermal treatments such as microwave ablation (MWA) have also been performed due to their several theoretical advantages such as a broader zone of active heating. These minimally invasive procedures offer a means to control tumor growth and improve survival by inducing thermal necrosis of cancerous tissues [7]. Despite these modalities being widely used, there is still ongoing debate regarding their comparative effectiveness and safety.

Previous research studies have compared the outcomes of such methods, with different results. A recent comparison suggested that MWA may offer superior advantages in terms of shorter procedure times and larger ablation zones with lower recurrence rates [8], while others have reported similar efficacy between the two modalities [9]. Furthermore, investigating these interventions is difficult due to improvements in technology and differences in healthcare delivery. A recent study found that RFA can lead to an increased risk of skin burns. However, there is no convincing evidence about the various clinical results, including local recurrence rates and survival rates [10].

It is worth noting that conducting systematic research is effective in synthesizing evidence from multiple studies to guide clinical decision-making. Pooling data from studies provides robust conclusions about the relative benefits and risks of different interventions. A comprehensive analysis comparing RFA and MWA is essential to determine the most fitting approach for managing inoperable tumors. Providing a detailed synthesis of the highest level of clinical evidence will help inform clinical practice and improve patient outcomes in the management of HCC. This review aims to rigorously evaluate the efficacy and safety of two common modalities in the management of HCC based on data from randomized controlled trials (RCTs).

## Review

Methodology

The study was conducted in alignment with the guidelines outlined in the Cochrane Handbook for Systematic Reviews of Interventions, version 6, and the findings were reported following the Preferred Reporting Items for Systematic Reviews and Meta-Analyses (PRISMA) standards [11].

Eligibility Criteria

This systematic review included only RCTs published in English at any time and compared the MWA and RFA therapies in HCC patients.

Participants

Studies that included patients with HCC.

Interventions

Direct comparison between MWA and RFA in managing patients with HCC.

Exclusion Criteria

We excluded duplicate articles, reviews, reports, observational cohort studies, single-arm studies, animal studies, and trials that lacked complete data.

Search Strategy

Online search was conducted using the following five databases: PubMed, CENTRAL, Medline, Ovid, and Scopus. We did not apply search filters, and the search encompassed the period from inception until August 1st, 2024. The search strategy incorporated the following MeSH terms: “Radiofrequency ablation,” “radiofrequency therapy,” “microwave therapy,” “microwave ablation therapy”, “hepatocellular carcinoma,” and “hepatic cancer.”

Selection of Studies

The processes of online search, screening the titles and abstracts, as well as revising the full text of relevant articles were conducted by two independent researchers. Any disagreements were resolved by consensus.

Data Extraction

We extracted the following data: (a) author details; (2) characteristics of the population (age, sample size, etc.), number of nodules, and duration of follow-up; and (3) the outcome measures.

Measured Outcomes

Primary outcome: The local tumor progression (LTP) and complete ablation (CA) were the primary outcomes.

Secondary outcome: The overall survival (OS) rate or any major complications were the secondary outcomes.

Quality Assessment of the Included Studies

We assessed the risk of bias (ROB) using the ROB2 tool for RCTs [12], as all included studies were found to be RCTs. The ROB2 tool comprises the following five domains: randomization, deviations from the assigned treatment, missing data, measurement of the outcome, and selective reporting of the outcomes and results. Moreover, the overall ROB is assessed by selecting the highest level of ROB out of the five domains. Robvis tool was used to visualize the figures [13].

Data Synthesis

Meta-analysis was performed using the Review Manager 5.3 (Cochrane Collaboration, UK). We evaluated the heterogeneity using Cochrane’s chi-square test, with a significance threshold set at 0.10, and the I^2^ statistic, where a value greater than 50% indicated significant heterogeneity [14]. We selected the random-effect model when significant heterogeneity was identified. However, we adopted the fixed-effect model when no significant heterogeneity was present. As our outcomes are dichotomous, the odds ratio (OR) was measured. Additionally, for this review, unadjusted p-values were employed for significance testing using a two-tailed cutoff of 0.05.

Results

Results of the Literature Search and Study Selection

**Figure 1:**
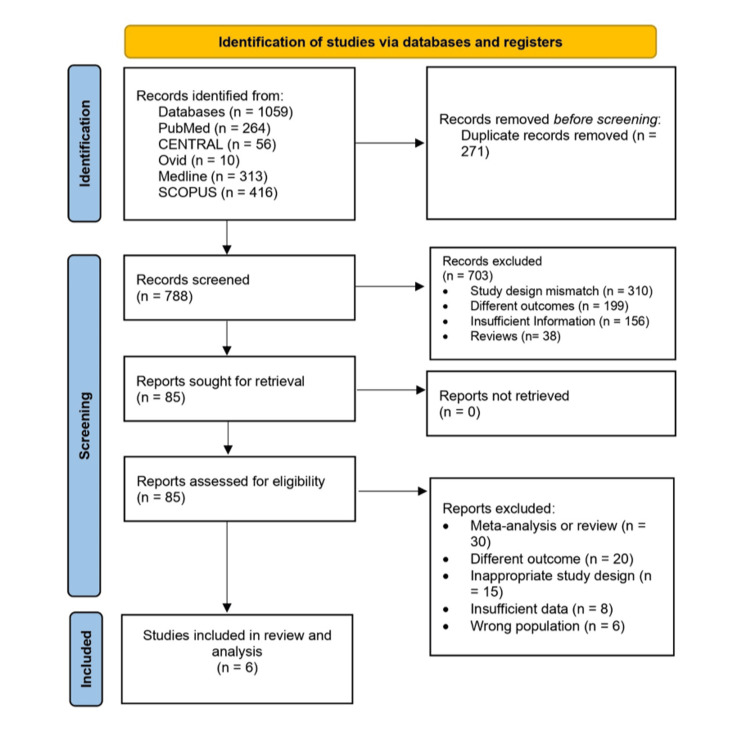
Preferred Reporting Items for Systematic Reviews and Meta-Analyses (PRISMA) flowchart of the included studies.

The search strategy yielded 1,059 records, of which 271 duplicates were removed. The remaining 788 records underwent screening of their titles and abstracts, and 703 records were excluded. The remaining 85 records were retrieved for full-text screening and were assessed for eligibility. Of these, 79 records were excluded. Finally, six studies were eligible for inclusion in the present review [15-20]. The details of the study selection are presented in the PRISMA flowchart (Figure 1).

Characteristics of the Included Studies

The six studies included were RCTs. Two studies were conducted in Egypt [15,18], and one study each was conducted in Switzerland [16], China [17], Italy [19], and Japan [20]. The total sample size of all trials was 826, ranging from 40 to 403 individuals, with ages ranging from 55 to 66 years across studies. The male proportion ranged from 69% to 84%. The enrollment duration ranged from 2002 to 2019. This systematic review addressed LTP as any new abnormal growth near or within the ablated area. CA was identified as the absence of enhancement in the ablated areas following the ablation procedure. OS was referred to as the duration from the initiation of ablation to either the date of death or the final follow-up. Major complications were classified as those of moderate or greater severity based on the Accordion Severity Grading System [21]. Table 1 presents the characteristics of the included studies.

**Table 1:** Characteristics of the RCTs included in the systematic review and meta-analysis. RCT: randomized controlled trial; RFA: radiofrequency ablation; MWA: microwave ablation

Study ID	Number of patients		Age (years)	Male (%)	Number of nodules	Follow-up		
	MWA	RFA			MWA	RFA	MWA	RFA
Kamal et al., 2019, Egypt [15]	28	28	55	77	34	34	12	12
Violi et al., 2018, Switzerland [16]	71	73	66	84	98	104	26	25
Yu et al., 2017, China [17]	203	200	NA	NA	265	251	35	35
Abdelaziz et al., 2014, Egypt [18]	66	45	55	71	76	52	40	40
Di Vece et al., 2014, Italy [19]	20	20	61	73	20	20	NA	NA
Shibata et al., 2002, Japan [20]	36	36	63	69	46	48	18	18

Risk of Bias Assessment

**Figure 2:**
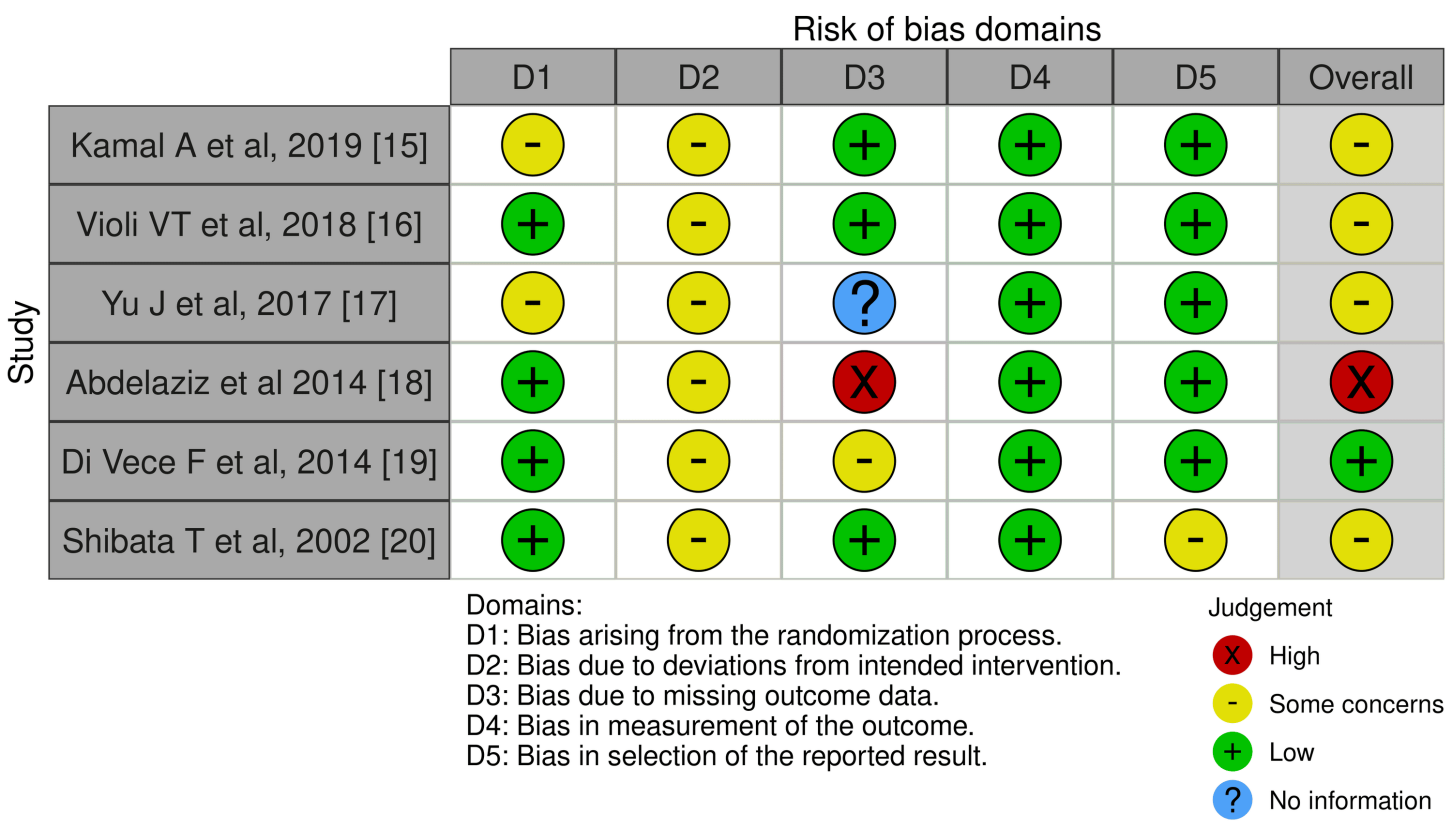
Risk of bias forest plots.

**Figure 3:**
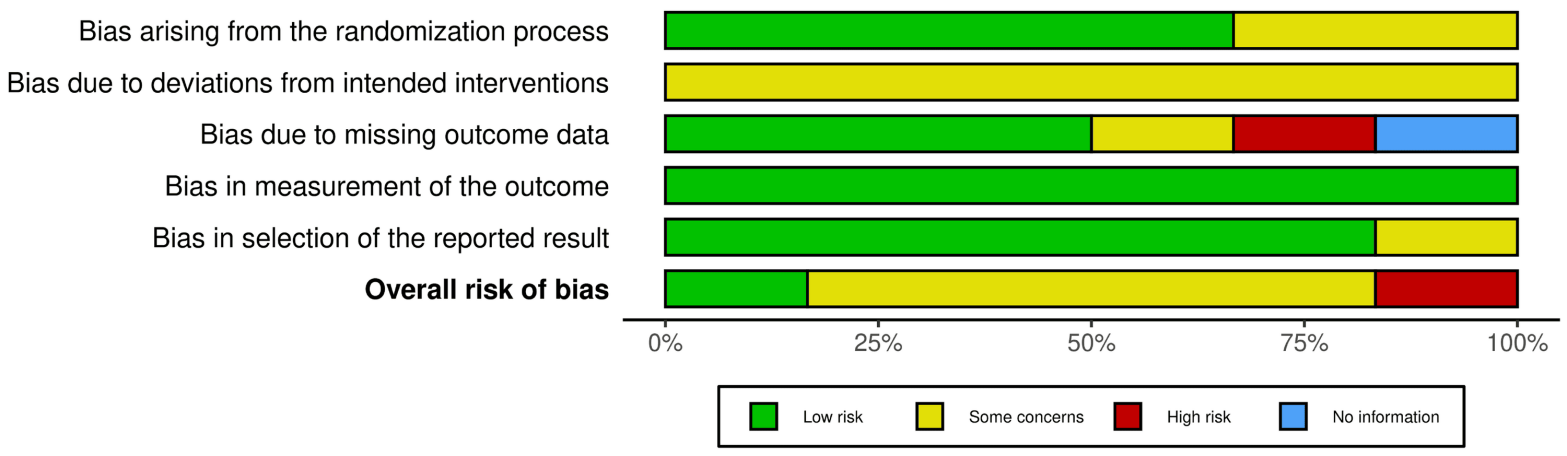
Risk of bias summary.

The ROB was assessed using the ROB2 tool for all included trials. The ROB in each domain and the overall risk are illustrated in Figure 2 and Figure 3. The ROB of four studies was low in terms of the randomization process [16,18-20], while two studies showed some concerns due to a lack of detailed information on the randomization process and allocation concealment [15,17]. In addition, one study showed a high risk of missing outcome data due to a high dropout rate (52.2%) [18]. Deviations from intended interventions were a concern in all studies [15-20] due to unclear information on the blinding of participants and personnel. The measurement of outcomes showed low ROB. Selective reporting of outcomes was low in two studies [16,17], but raised some concerns in the other four studies [15,18,19,20], due to the non-availability of a pre-registered protocol that was finalized before performing the study to compare the methodology.

Results of the Meta-Analysis

**Figure 4:**
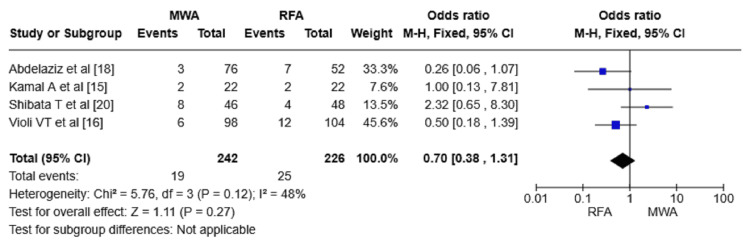
Forest plot comparing LTP between MWA and RFA. LTP: local tumor progression; RFA: radiofrequency ablation; MWA: microwave ablation; M-H: Mantel-Haenszel method

Local tumor progression: The LTP was reported in four RCTs, with 468 patients. MWA exhibited a lower LTP rate compared to RFA in the selected studies (OR = 0.70, 95% confidence interval (CI) = 0.38-1.31, p = 0.27). No significant heterogeneity was found between RCTs (I^2^ = 48%) (Figure 4).

**Figure 5:**
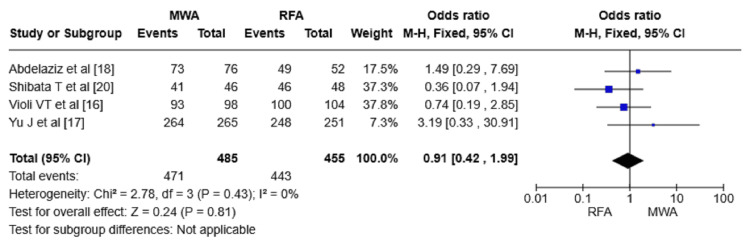
Forest plot comparing the CA rate between MWA and RFA. CA: complete ablation; RFA: radiofrequency ablation; MWA: microwave ablation; M-H: Mantel-Haenszel method

Complete ablation rate: Four RCTs involving 940 patients reported the rate of CA. The RFA group experienced a lower event rate compared to the other group (455 versus 485). Nevertheless, the difference in CA rates between MWA and RFA groups in these RCTs was not statistically significant (OR = 0.91, 95% CI = 0.42-1.99, p = 0.81) (Figure 5).

**Figure 6:**
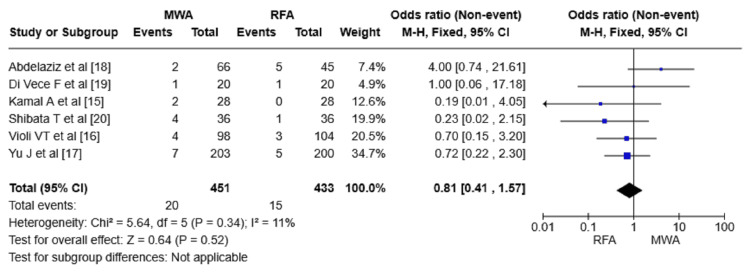
Forest plot comparing major complications. RFA: radiofrequency ablation; MWA: microwave ablation; M-H: Mantel-Haenszel method

Major complications: All six RCTs were analyzed for major complication rates among the MWA and RFA groups. The total number of patients included was 884, with no significant heterogeneity in each of the six RCTs (I^2^ = 11%). The meta-analysis revealed major complications for MWA and RFA, with rates of 20 and 15, respectively, suggesting no significant difference between the two (OR = 0.81 95% CI = 0.41-1.57, p = 0.52) (Figure 6).

Overall survival: The included trials reported OS rates at different time intervals, such as one-year, two-year, and three-year intervals, but we could not pool the results due to the heterogeneity in the timing of the reported data. However, the results of all studies showed no statistical significance between the different reported rates. For instance, Violi et al. [16] found no significant difference in median time to progression or OS over a two-year follow-up, with the overall actuarial probability of survival being 91.6% at one year and 86.1% at two years. Abdelaziz et al. observed higher actuarial probabilities of survival at one and two years for patients treated with MWA (96.4% and 62%, respectively) compared to those treated with RFA (67.6% and 47.4%, respectively) [18], although the differences were not statistically significant. In addition, Yu et al. reported one-year, three-year, and five-year OS rates of 96.4%, 81.9%, and 67.3% for MWA versus 95.9%, 81.4%, and 72.7% for RFA (p = 0.91). However, Kamal et al. did not report the OS rates but found that local tumor recurrence-free survival at one year was estimated to be 90.9% in the RFA patients and 92.3% in MWA patients (p = 0.932) [15].

Discussion

This review incorporated six RCTs, including 826 patients, and provided a comparison of two widely used ablative techniques regarding their LTP, CA, complication rates, and OS rates. Our findings revealed that MWA resulted in lower LTP and higher CA rates. However, the effect of complications was higher in the MWA therapy group. Despite that, the differences between all parameters were not significant.

Tumor ablation is an alternative to surgery used for treating tumors in the liver, kidney, bone, and lung. This involves heating or cooling tissue through thermal energy so that it kills cancerous cells (about −40°C or above 60°C). Different tumor ablation methods are applied globally which include the most common modalities: radiofrequency and microwave. RFA uses a needle electrode passed into the tumor and an alternating current to produce heat by ion agitation causing coagulative necrosis. MWA, on the other hand, employs a microwave antenna that expels electromagnetic waves that oscillate water molecules rapidly leading to friction and heat resulting in cell death. The main difference is that RFA uses electrical currents to generate heat while MWA uses electromagnet waves, thus producing higher temperatures over larger areas very fast [22].

In this analysis, MWA presented a lower LTP rate compared to RFA, suggesting a higher benefit of MWA in stopping tumor recurrence. However, the OR for LTP was 0.70 (95% CI = 0.38-1.31, p = 0.27), indicating the non-significant difference between the two modalities. This finding is aligned with previous research by Ding et al. (2013), which also reported lower LTP rates for MWA compared to RFA in the treatment of HCC [23]. In addition, Lee et al. conducted a real-world comparative study and observed that MWA had a higher efficiency and lower local recurrence rate compared to RFA in both treatment-naïve and recurrent HCC patients [24]. Similarly, Gupta et al. conducted a network meta-analysis, which found that the local recurrence rate was lower in MWA compared to RFA and cryoablation, suggesting a superiority of MWA in achieving complete and durable tumor control [25].

Regarding CA rates, our analysis found no significance between MWA and RFA (OR = 0.91, 95% CI = 0.42-1.99, p = 0.81). This finding suggests that both techniques are effective in complete tumor ablation. Similarly, Han et al. (2020) concluded that both addressed modalities have comparable CA rates in the treatment of early-stage HCC [26]. However, Santambrogio et al. compared laparoscopic MWA and RFA for small HCC (≤3 cm) and found that MWA was associated with a higher efficiency in achieving complete response [27]. Gaia et al. (2021) reported that MWA was more efficient than RFA in terms of complete response among cirrhotic patients with early HCC [28].

Furthermore, the safety profile of both modalities was also similar because there was no significance in the major complications (OR = 0.81, 95% CI = 0.41-1.57, p = 0.52, I^2^ = 11%). This indicates that both procedures have low rates of serious adverse events or any other type of risk to warrant calling them safe and well-tolerated by patients. This finding is aligned with the study by Chong et al. (2020), which reported that major complication rates between MWA and RFA lack significant differences [29].

The findings of this meta-analysis have important insights for clinical practice that should be further validated and assessed. The lower LTP rate associated with MWA suggests that it may be the preferred option for patients with liver malignancies, particularly in cases where reducing the risk of recurrence is significant. The comparable CA rates and safety profiles indicate that both MWA and RFA are effective and safe. This provides healthcare providers with flexible treatment approaches that are customized to patient-specific factors and available resources.

The review included only RCTs which improves the quality of the study. This helps improve the reliability and validity of the results. In addition, implanting a comprehensive detailed search strategy and accurate assessment of study ROB further strengthen the conclusions.

However, several limitations should be acknowledged. First, the number of selected studies was relatively small, and the sample sizes within individual studies varied considerably. This could affect the generalizability of the results. Second, the heterogeneity in reporting OS data prevented a pooled analysis of this important outcome. In addition, another limitation was the difference in patient demographics in different studies and different races. Future studies should aim to standardize the reporting of survival metrics to enable more robust comparisons.

Additionally, some studies exhibited concerns regarding the ROB, particularly related to randomization processes, allocation concealment, and blinding of participants and personnel. Addressing these methodological issues in future trials will be crucial for ensuring more reliable and unbiased results.

Additional analysis is required to verify the durability of MWA compared to RFA. There is a need for large-scale multicenter randomized RCTs with standardized outcome measures and longer follow-up durations that will generate more conclusive results. Furthermore, comparing the cost-effectiveness of the two studied modalities would give insights into healthcare provider settings and help inform policy decisions.

## Conclusions

This study suggests that MWA may offer a lower risk of LTP compared to RFA, with similar rates of CA and major complications. Both techniques appear to be effective and safe for managing liver malignancies, providing clinicians with valuable options for personalized patient care. Further high-quality research is needed to confirm these findings and guide clinical decision-making.
